# Priming With Silicon: A Review of a Promising Tool to Improve Micronutrient Deficiency Symptoms

**DOI:** 10.3389/fpls.2022.840770

**Published:** 2022-03-01

**Authors:** Lourdes Hernandez-Apaolaza

**Affiliations:** Department of Agricultural Chemistry and Food Science, Universidad Autónoma de Madrid, Madrid, Spain

**Keywords:** silicon, priming, stress memory, plant recovery, micronutrient deficiency

## Abstract

Priming consists of a short pretreatment or preconditioning of seeds or seedlings with different types of primers (biological, chemical, or physical), which activates various mechanisms that improve plant vigor. In addition, stress responses are also upregulated with priming, obtaining plant phenotypes more tolerant to stress. As priming is thought to create a memory in plants, it is impairing a better resilience against stress situations. In today’s world and due to climatic change, almost all plants encounter stresses with different severity. Lots of these stresses are relevant to biotic phenomena, but lots of them are also relevant to abiotic ones. In both these two conditions, silicon application has strong and positive effects when used as a priming agent. Several Si seed priming experiments have been performed to cope with several abiotic stresses (drought, salinity, alkaline stress), and Si primers have been used in non-stress situations to increase seed or seedlings vigor, but few has been done in the field of plant recovery with Si after a stress situation, although promising results have been referenced in the scarce literature. This review pointed out that Si could be successfully used in seed priming under optimal conditions (increased seed vigor), to cope with several stresses and also to recover plants from stressful situations more rapidly, and open a promising research topic to investigate, as priming is not an expensive technique and is easy to introduce by growers.

## Introduction

According to the National Climate Assessment (NCA)-USDA, the highest losses in global crop production can be attributed to abiotic stresses (∼50%), followed by weeds, insects, and pathogens (Srivastava et al., 2021). To cope with biotic and abiotic stresses, and due to their sessile life, plants have developed a great variety of adaptation strategies to mitigate their stressor effects and to survive in such stress conditions. These strategies are especially relevant to fight against climate changes that crops should afford, which significantly affect biotic and abiotic stressors (pests, drought, salinity, etc.) or nutrient imbalances (deficiencies or toxicities).

### What Priming Means? General Concepts

The plant stress responses should be first divided into two different approaches: acclimatation and priming. Acclimation is referred to plant strategies induced to cope with long periods of stress duration, to which the metabolism of plants, with more or less success, will be adjusted (Wiszniewska, 2021), maintaining higher amounts of stress-protective compounds and therefore being prepared for future stress episodes. On the contrary, priming is defined as the stimulation of a specific physiological state that allows plants to give a stronger and rapid response against stress compared with plants without priming (Balmer et al., 2015), which is like a vaccine. Usually, priming is carried out by short pretreatment or preconditioning with different compounds (chemical, biological) or by altering physical factors for a determined period (Filippou et al., 2012; Leuendorf et al., 2020). Most of the primers are used to affect the synchronization of seed germination and give plants a better resistance against stressful conditions (Srivastava et al., 2021). Primer treatments in seeds activate various enzymes such as proteases, dehydrogenases, hydrolases, and α-amylase, and this weakens endosperm and mobilizes reserve substances, which finally improve seed vigor. In addition, DNA repair proteins, stress-responsive transcription factors, and metabolites such as antioxidants, osmolytes, and sugars are upregulated, which contributes to performing phenotypes more tolerant to stress in the primed plants (Farooq et al., 2019). It is also considered that priming promotes the development of stress memory in plants, which improves plant resilience against adverse conditions (Savvides et al., 2016). Priming effects can last for the complete growth cycle of the plant or several generations, although priming is given only during the initial seed germination or at the seedling stages and has a short duration (hours for seeds or a few weeks for seedlings).

There is increasing evidence that not only animals but also organisms without a specific nervous system (plants, fungi, or bacteria) can “remember” a past event (i.e., Thellier and Lüttge, 2013). This memory may shape or “prime” future responses to environmental conditions, which gives a stimulus-dependent and phenotypic plasticity of response traits. So, the environmental adaptation of an individual by adjusting its physiological or developmental phenotype is mediated by such plasticity (Sultan, 2000; West-Eberhard, 2003).

Induction of direct defenses can minimize the benefits of enhanced protection because this is usually correlated with high physiological expenses. By contrast, primed plants are almost equally protected but need considerably lower fitness costs (van Hulten et al., 2006; Wang et al., 2015). This makes priming-based approaches valuable to cope with several biotic and abiotic stresses. Due to its resource-saving character, priming is considered advantageous over acclimation (Leuendorf et al., 2020).

Pastor et al. (2014) and Balmer et al. (2015) defined different states in a priming process: (1) “Priming state” achieved after the application of the priming stimulus and lasts until plant exposure to stress. During this period, the levels of various primary and secondary metabolites, hormones, enzymes, and other molecules are slightly altered (tricarboxylic acid cycle metabolites; amino acids, sugars, reactive oxygen species (ROS), pathogenesis-related proteins (PRPs), salicylic acid), placing the plant in a standby state. (2) “Postchallenge primed state” appears when the plant becomes stressed, and the plants rapidly induced the corresponding reactions to fight the stressor. (3) “Transgenerational primed state” observed in plants that have a priming memory due to become from seeds obtained from primed parental plants.

It is generally assumed that priming acts on the phenotype of individuals, and its effects are attributed to epigenetic, hormonal, cellular, and other phenotypic changes (Hilker et al., 2016). A clear link between changes in protein synthesis or gene expression and alterations in phenotype is not usually observed (Balmer et al., 2015). For that reason, it is considered reversible, because the applied stimulus apparently keeps the DNA sequence unchanged. So, priming allows for reversion to the original state. During the poststress phases, the primed plant behavior is influenced by factors such as developmental stage or environmental conditions and strongly depends on the plant–stressor combination (Balmer et al., 2015). The readjustment of an organism from a primed to inexperienced state, that is, the “forgetting” of the priming event, may be dependent on the lifetime of the cellular marks left by priming that are called upon exposure to stress (Hilker et al., 2016).

For activating the priming process, the priming agent and the stressor could be of the same nature; for instance, Ding et al. (2012) described that multiple exposures to drought “train” Arabidopsis responses to coping with this abiotic stress. Many other examples for abiotic and biotic stresses of this type are referred to in Hilker et al. (2016), like pathogen infection primes plant resistance against future pathogen infection. On the other hand, the priming agent and the stressor could have a different nature. Several studies have demonstrated that exogenous applications of H_2_O_2_ induce tolerance to drought, high temperatures, chilling and salinity, and also heavy metal stresses (Hossain et al., 2015). This could be explained by the primer action through the activation of general antioxidant and signaling pathways.

Priming can be applied at various developmental stages of the plant life cycle and to various plant parts. The most used is seed priming because its simplicity allows a wide utilization of this technique. Seed priming is a presowing treatment that exposes seeds to ascertain a solution for a certain period that allows partial hydration, but germination does not occur, because the moisture of the seed is not enough to cause the seed to germinate. However, this level is enough to start many of the physiological processes associated with the early phase of germination (pregermination metabolism) (Ibrahim, 2016). After priming, seeds are redried to their initial moisture content to allow the storage of the primed seeds (Di Girolamo and Barbanti, 2012) or could be directly sown. The optimization of the priming process is necessary, due to lots of factors that can affect the seed response to primers, such as duration, temperature, seed vigor, plant species, and storage condition. There have been different reports about the positive effects of priming in plants. In general, primed seeds present improved vigor, reduction in germination time with changes in molecular, cellular, physiological, and biochemical aspects and result in higher seed vigor and a better crop establishment and yield of crops (Yacoubi et al., 2011; Di Girolamo and Barbanti, 2012; Finch-Savage and Bassel, 2016). These changes include cell division and elongation, plasma membrane fluidity, the induction of stress-responsive proteins, changes in transcriptome and proteome, H^+^-ATPase activity (Soeda et al., 2005; Zhuo et al., 2009; Kubala et al., 2015; Finch-Savage and Bassel, 2016), and changes in the antioxidant system activity (Chen and Arora, 2011; Kubala et al., 2015). Moreover, priming will facilitate the seeds to cope with environmental stresses during seedling establishment and show increased stress tolerance at the whole-plant level (Yacoubi et al., 2011; Srivastava et al., 2021), because, during the dehydration and soaking steps, it may generate moderate abiotic stress (Ashraf and Foolad, 2005) probably due to the radicle protrusion repression. Plants will remember such stress and their memory phase is long term, which ranges from weeks to months (Srivastava et al., 2021), and remember that seed priming is applied only once. Similarly, during soaking, seeds bind water and absorb protective and biologically active compounds (Wiszniewska, 2021). Beneficial effects are then expressed in developing seedlings and increased plant vigor and survivability under biotic or abiotic stresses.

Less often, priming is applied to seedlings or their parts, and also young plants in active growth phases. This approach is focused on remediation of plant stresses in specific plant regions or organs. For example, to cope with metal toxic levels using phytohormone priming treatments, which application to seedlings and developed plants are preferred than to seeds (Sytar et al., 2019). Moreover, priming can be applied *in vitro* culture to organs, or their fragments excised from donor plants (Wiszniewska, 2021). As a result of prolonged selection in the presence of stress agents, such as salinity, for example, the plant cells’ behavior and the regenerated plantlets are modified when compared with plants without a priming treatment (Chun et al., 2019).

A well-nourished plant that is supplied with enough and complete essential nutrients diet will have sufficient and more energy and metabolic resources to invest in memory stress responses than a plant grown with not all the nutrients or with a low amount of them. The same for other environmental conditions influences plant development, such as light, humidity, oxygen, etc. Defense strategies impose a substantial demand for resources, which reduce growth (Huot et al., 2014) and could decrease photosynthesis, which means a reduction of energy reserves. So, a balance between nutrition, growth, and defense should be achieved to optimize plant wellness.

Moreover, how much an organism invests in priming depends on the age at which experiences this stimulus (Hilker et al., 2016). After priming, short and long-term stress memories could be activated. The first should work to cope with imminent stress and the second with future stressors. The different plant costs of these two strategies will be the key point of the process, and plant age plays an important role in it, as the plant that expends the costs of long-term memory also needs to maintain enough resources for growth and plant protection. Mechanistic differences between these types of memories and if they are different approaches or depend on plant species or age or even nutritional factors require further study. In general, young plants could benefit from a long-term stress memory, and adult plants are expected to invest in short-term memory, to have resources for its reproduction (Huot et al., 2014).

### Stress Memory and Priming

Srivastava et al. (2021) considered that seed priming and stress memory are the two faces of the same coin. These authors proposed that primers generate a mild (sublethal) stress inside the seed that prepares the future seedlings to cope with stresses more efficiently than unprimed plants. This fact was based on the observation that the growth of seedlings from primed seeds is slower than that of non-primed seeds a few hours after postpriming. It could be said that seed priming forces plants to begin germination under stress, which will create a stress memory on the seeds. Several stress marks can be imprinted on the seed genome just as in stress-primed plants, which lead to improved stress tolerance. However, in the case of seedlings or plant priming, the stress memory was created specifically in the plant.

Plants have to cope with different stresses all along their crop cycle and retain “memories” of previously encountered stresses as an adaptive mechanism that helps them to encounter forthcoming stresses more rapidly and efficiently. These memories are called “acquired tolerance.” They can produce a short-term effect (somatic memory), a memory that can be transmitted to succeeding generations (intergenerational memory), or, in some cases, a memory that can be inherited across generations (transgenerational memory). Such memories can be induced artificially through preexposure to a low-dose stressor or by the addition of beneficial compounds such as silicon, or by natural exposure to recurrent stress episodes (e.g., stational drought) (Srivastava et al., 2021). “Somatic memory” in plants has been explained through several mechanisms that include chromatin remodeling, alternative transcript splicing, metabolite accumulation, and autophagy. However, chromatin-dependent regulation is considered as a key mechanism for regulating stress memories (Bäurle, 2018; Friedrich et al., 2019; Bäurle and Trindade, 2020). Chromatin could be defined as a substance within a chromosome consisting of DNA and proteins, being histones the most common proteins in chromatin. Proteins help package the DNA in a compact form that fits in the cell nucleus. Moreover, DNA replication and gene expression are associated with changes in chromatin structure. At the chromatin level, stress memory is granted by different epigenetic modifications, which alter the overall accessibility of genes for transcription. The deposition of active histone marks (cellular marks) is known to be regulated by stress situations. As mentioned, other mechanisms, independent from changes in chromatin, also regulate stress-induced somatic memory in plants. The accumulation of cellular metabolites attributed to stressful situations can also modulate plant responses during the memory phase. Moreover, the adjustment of physical properties, such as building a thicker cell wall with higher lignin content by salt primed cells of *A. thaliana*, is important for regulating salt stress-induced memory (Chun et al., 2019). Finally, autophagy in plants plays an opposite role because it degrades stress-induced proteins and other biomolecules during the recovery phase (Su et al., 2020), so this process could act as a negative regulator of stress-induced memory.

Epigenetics studies changes in genes’ work due to the environment. Epigenetic changes are reversible (unlike genetic changes) and do not change the DNA sequence, but they can change how a plant reads a DNA sequence. These characteristics of epigenetic changes give a dynamic and persistent stress response mechanism by which a gene, or a network, is activated to cope with a stress situation. When the stressor stopped, two options are possible, and it reverses to the initial state or keeps a cellular stress mark to facilitate a more potent and quicker answer to future stresses. Epigenetics includes a heritable (mitotic or meiotic) component that allows preserving this mark between generations (Ding et al., 2012). Therefore, two types of cellular marks could be distinguished, the first associated with the stressor presence, but removed when the stress situation finish, called a chromatin mark and the second, which implies the persistence of the chromatin mark after the disappearance of the stressor, called an epigenetic mark (Ding et al., 2012). This second type of marks are related to the “transcriptional memory,” which could be defined as a type of information that persists after the plant’s recovery from the stress and which influences subsequent transcriptional responses (Ding et al., 2012). These authors described the transcriptional memory of *Arabidopsis* plants after applying successive dehydration–watered treatments in a relatively short time. They observed that *Arabidopsis* leaf cells’ ability to retain water was altered during repetitive exposures to dehydration stress, and this observation was sustained by increased rates of transcription and elevated transcript levels from a subset of the stress–response genes (trainable genes). Two different marks were found only at the trainable genes during their recovery period, and therefore, transcriptional memory was associated with them. The marks were as follows: high levels of trimethylated histone H3 Lys4 (H3K4me3) nucleosomes and the presence of Ser5P polymerase II (serine 5 phosphorylated Polymerase II) (the transcription initiation form of RNA polymerase II). At the trainable genes, the persistence of H3K4me3 and Ser5P polymerase II marks was related to the transcriptional memory length. In contrast, in the non-trainable genes, these two marks increase during stress but were reduced to basal levels when the stress was eliminated, and the plants try to recover from this situation. In diverse plant species and tissue cultures, changes in histones, including H3K4me3, in trainable genes have been reported when studied several abiotic stresses such as salinity, low temperatures, hypoxia, and drought (Sokol et al., 2007; Kim et al., 2008). Likewise, stress-induced memory has been reported in various crops that include sugarcane (Marcos et al., 2018), rice (Li et al., 2019), maize (Virlouvet et al., 2018), and wheat (Wang et al., 2020), apart from model plant *A. thaliana*.

Moreover, there are some reports concerning crosstolerance, crossresistance, or crossprotection, which means that the stress response mechanism activated in a plant to cope with specific stress could have a beneficial effect on the plants when a different stress situation appears. This is a similar mechanism that the one observed in several pests against pesticides, and which severely minimized their efficacy. This has been observed, for instance, in the case of pepper (*Capsicum annuum* L.). When grown in an excess of Cu, severe stress was caused in the plants, and several responses were induced to mitigate it, but a decrease in the disease symptoms generated by the inoculation of *Verticillium dahlia* was obtained (Chmielowska et al., 2010).

### Plant Stress Recovery

Dealing with recurrent stress situations is a key point in plant memory. In such situations, plants could reduce their response under recurrent stimulus or present a positive and reinforced response to the stressor (see Table 1 for a summary of the different memory types). The most important thing to consider in a recovery situation is that the previously caused damage needs to be repaired. Then, the plant needs to continue its growth. As mentioned before, plants have the ability to remember previous stress by maintaining some cellular marks that prepare them for developing a better strategy to cope with future stresses. But these responses are frequently accompanied by a growth reduction (Huot et al., 2014). Therefore, after the stress, an efficient and quick recovery with a reversion of the stress changes is necessary to obtain the maximal growth and reproduction rates under the new conditions. In this situation, plants must balance the necessary recovery and maintenance of stress memory to cope with future stresses. For this purpose, autophagy plays an important role in regulating recovery from stress, by eliminating compounds and cellular marks not currently needed and resetting cellular memory (Thirumalaikumar et al., 2021).

**TABLE 1:** A comparative among seed priming, somatic stress memory, and effect on resupply or restoration of optimal conditions in plants.

	Seed priming	Stress memory	Resupply
Application	Before seed germination	Seedlings/Plants	Seedlings/Plants
Number of applications	One	Every sublethal stress situation	After stress
Memory phase	Long term (weeks-months)	Short-term (hours-days)	Under investigation
Primers	Not necessary to be the same as future stress	The same as future stress (exception cross-tolerance)	Elimination of the stressor
Mechanism	Under investigation	Chromatin modifications (trainable genes), metabolite accumulation, etc.	Under investigation (trainable genes with sustained expression during recovery?)
Stress response	Improved	Improved	No stress
Non-stress situation	Possible plant growth reduction	Possible plant growth reduction	Total/partial recovery

In that way, plants submitted to several stressors are reported to maintain stress memory when the stress disappears, due to memory of trainable genes. Based on their transcriptional profile, Bäurle (2018) classified them into following types: type I genes that are upregulated upon first stress and show sustained expression during the recovery phase; and type II-genes that are induced upon first stress and hyperinduced upon recurrent stress separated by a few days or weeks of recovery under stress-free conditions, but without sustained expression during recovery. However, the mechanisms that control the regulation of these genes, and which characteristics distinguish them from non-trainable genes, need further research. Marcos et al. (2018) observed that sugarcane plants that have suffered a water deficit improved their responses when submitted to a new drought event. Even more, when rewatered, they used water with more efficiency than those plants grown with an optimal irrigation pattern. This suggests that sugarcane plants present stress memory under varying water availability and is a clear example of trainable genes of type I. The same was previously observed by Ding et al. (2012). A rapid increase in a specific abscisic acid (ABA) transporter expression and distribution and an increase in the endogenous ABA content were obtained under drought and probably under other stresses. This hormone plays a key role in the plant stress resistance and changed the postresponse gene type into the memory gene type. This improved the tolerance to following stress episodes and also the recovery capacity of the plant. Qin et al. (2021) in Arabidopsis plants submitted to different drought–water periods observed that during a second recovery period, the ABA transporter expression level and ABA content were higher than at the first recovery.

After metal stress conditions, plant growth recovery depends on different factors such as the applied metal, stress intensity, and duration, and plant species. For example, soybean seedlings were able to restore their growth during 7 days of recovery, after 2 days under high Cd concentration (89 and 223 μM Cd). They showed the same levels of chlorophyll and photosynthetic parameters as the control. The only significant difference was the shortening of the roots in plants previously treated with Cd (Holubek et al., 2020). Although, in a similar study with tobacco suspension cells treated with 50 μM Cd, a fully restored growth was obtained when treated for 3 days but not when stress conditions were prolonged only 1 day more (Fojtova, 2002). Similarly, the recovery of Arabidopsis after 21 days of phosphate deficiency was studied, and after just 1 day of P resupply, the expression of 40% of induced genes was reversed and 80% after 3 days. This latter corresponded to the reestablishment of the adequate root phosphate concentration. However, after 31 days of resupply, 80 genes remained differentially regulated, and the reversion of chromatin states during recovery (Secco et al., 2015). Plant recovery from Fe deficiency was tested in strawberry (a very susceptible plant to this stress), in which plants were grown initially with two Fe concentrations (0 or 10 μM), and then, half of the plants growing without Fe were Fe-resupplied by adding 10 μM Fe (Gama et al., 2016). These authors concluded that Fe stress does not induce permanent damages in the photosynthetic apparatus, as they observed a complete regreening of Fe resupply plants. The rapid response to the resupply of iron (12 days) has been assigned to quick access of Fe *via* the xylem to young leaves (Pestana et al., 2012). This also leads to significant biomass recovery, although, as expected, resupply plants were smaller than plants with optimal Fe nutrition. After Fe resupply, there was a boost of Fe reduction (an increase of Fe chelate reductase). Thus, explaining the high Fe contents in flowers and similar content in the rest of the organs of the recovered plants is compared with well-fed plants. Also, the Fe distribution in plants was altered in resupply plants, whereas Fe-sufficient plants accumulated Fe mainly in mature leaves, but recovered plants mobilized Fe to flowers (future fruits). All these facts may be related to modifications in trainable genes that persist after stress suppression (type I genes) and are reflected in Table 1.

## Priming With Silicon

Although Si’s essentiality for plant metabolism has not been proved yet, the sustainability of the production of several crops, such as rice or sugarcane, depends on this element. Chemical speciation and amount of Si in the soil affect the absorption taken place by plants, being silicic acid (H_4_SiO_4_), the form of Si which is absorbed. Silicon dioxide constitutes 50–70% of the mass of the soil (Ma and Yamaji, 2006), and clay minerals and sand are the most important soil components with silicon on their structure. Weathering, as a natural phenomenon, causes the release of Si into the soil solution and provides Si to plants; however, intensive cropping contributes to a Si depletion in the soils. The knowledge on the Si effect on the mitigation of biotic and abiotic stresses and the Si reduction in soils make Si fertilizers application a relevant agricultural practice. Generally, two aspects of Si effects are considered: (1) The usefulness level of Si application varies in different plant species: beneficial effects are usually more obvious in plants that accumulate high levels of Si in their shoots (see an example in Gonzalo et al., 2013); and (2) The positive and multilateral effect of Si is more observable when the plant is under stress or in the recovering process from this stress (i.e., Bityutskii et al., 2014; Carrasco-Gil et al., 2018; Nikolic et al., 2019; Hernandez-Apaolaza et al., 2020; Thorne et al., 2020; Arafa et al., 2021; Martín-Esquinas and Hernández-Apaolaza, 2021).

In plants, the polymerization of Si in the intercellular spaces and beneath the leaf’s cuticles due to its accumulation in shoot creates a physical barrier against pathogen attack and contributes to maintaining plant erectness, with the subsequent improvement of photosynthesis. Moreover, the Si in the root apoplast contributes to reducing some nutrient–contaminants translocation to shoot and activates various metabolic pathways. These physical and biochemical combination enhances plant defenses against abiotic (drought, salinity, nutrient imbalances, etc.) and biotic stresses (insects, fungus, and bacteria). In addition, soluble Si in the plant system attracts beneficial predators and parasitoids during pest attacks and consequently increases biological control (Bakhat et al., 2018). An example of the beneficial effect of priming with Si against an abiotic stress situation (see Table 2), such as drought, was given by Hameed et al. (2021), who observed the improvement in wheat yield when seeds were primed with Si, by inducing a priming memory in seeds that increased drought tolerance during seed germination, seedling growth, and plant developmental stages. In the same way, Si has been used as a primer to minimize metal toxicity (Abd_Allah et al., 2019) in mustard (*Brassica juncea*) seedlings under Ni toxic exposure. In this experiment, after 1 week of Ni treatment, plants (18-day-old seedlings) were primed with 10^–5^ M Si as Na_2_SiO_3_ added to the nutrient solution, for 1 week, and finally, they were collected after 2 weeks more. They observed that Si decreased root to shoot Ni translocation and upregulate enzymes associated with antioxidant defense, glyoxalase detoxification systems, and also a sufficient primary and secondary osmoprotectant accumulation, which ameliorated Ni toxic symptoms in this crop. Another example of the beneficial effect of Si priming is given under alkaline stress conditions (Liu et al., 2018). In this research, 30-day-old alfalfa seedlings were primed with 0 or 2.25 mM Na_2_SiO_3_⋅9H_2_O during 36 h, and then, plants were stressed for 48 h by adding 25 mM Na_2_CO_3_ to the nutrient solution. It has been obtained that Si priming significantly alleviated the damage symptoms and greatly increased the chlorophyll content of stressed plants. Although the Si treatment did not show appreciable benefits under unstressed conditions, which indicates that the Si priming effect was specific to alkaline-stressed plants. Moreover, it altered the root morphology of alfalfa seedlings, which enhanced the uptake ability of the roots to uptake nutrients and water, and significantly increased root dry weight, decreasing membrane injury and malondialdehyde content, and increasing antioxidant enzyme activities. Furthermore, Si priming significantly decreased Na accumulation and increased K accumulation in the leaves under alkaline stress. Meanwhile, Si priming decreased the accumulation of metal ions such as Mg, Fe, Mn, and Zn in the roots of alfalfa seedlings under alkaline stress.

**TABLE 2:** Different Si sources use for priming and control abiotic stresses.

Priming agent	[Si]	Stress	Priming period (h)	Crop	References
	
	Seed priming				
Sodium silicate	0, 10, 20, 30, 40, 50 mM	Salinity	8	Wheat	Azeem et al., 2015
	1.5 mM	Alkaline	12	Maize	Abdel Latef and Tran, 2016
	20,40, 60 mM	Drought	8	Wheat	Hameed et al., 2021
Sodium silicate nano	0, 300, 600, 900, 1,200 mg/L	Cd toxicity	20	Wheat	Hussain et al., 2019
Nanosilicon (nSiO_2_)	1.66, 6.65, 13.3, 19.97, 26.63 mM	None	4	Maize	Kumaraswamy et al., 2021
	0.2, 0.4, 0.6, 0.8, 1, 1.2 mM	None	8	Sunflower	Janmohammadi and Sabaghnia, 2015
	0, 100, 500 mg/L	None	24	Lemon balm	Hatami et al., 2021
SiO_2_	0.01% w/v	None	4	Maize	Kumaraswamy et al., 2021
	0.5, 1.0, 1.5%	Drought	6	Wheat	Ahmed et al., 2016
	3% 3.5% w/v	Drought	8	Rice	Ali et al., 2021
Silicic acid	0.5, 1.0, 1.5%	Drought	6	Wheat	Ahmed et al., 2016
	0, 0.063, 0.125, 0.25, 0.5 mM	Drought	24	Tomato	Chakma et al., 2021
3-aminopropyl triethoxy silane (pH 5.95)	0, 5, 10, 15, 20, 25 g⋅L-1	None		Maize	Sun et al., 2021
3-glycidoxypropyl trimethoxy silane (pH 9.42)	0, 5, 10, 15, 20, 25 g⋅L-1	None		Maize	Sun et al., 2021

	**Seedling priming**				
	
	**[Si]** **mM**	**Stress**	**Priming period (weeks)**	**Crop**	**References**

Sodium silicate	0.0, 0.5, 1.0	Fe deficiency	2	Soybean	Gonzalo et al., 2013
	0.0, 0.5, 1.0	Fe deficiency	2	Cucumber	Gonzalo et al., 2013
	0.0, 0.5, 1.0	Zn deficiency	2	Soybean	Pascual et al., 2016
	0.0, 2.25	Alkaline	36 h	Alfalfa	Liu et al., 2018
	0.01	Ni toxicity	1	Mustard	Abd_Allah et al., 2019

	**Plant recovery**				
	
	**[Si]** **mM**	**Stress**	**Recovery time**	**Crop**	**References**

Silicic acid	0.0, 1.5	Fe deficiency	5 days	Cucumber	Hernandez-Apaolaza et al., 2020
	0.0, 1.5	Zn deficiency	11 days	Cucumber	Lozano-González et al., 2021
Potassium silicate	0.0, 1.8, 3.6	Hyperhydricity	2 weeks	Carnation	Soundararajan et al., 2017
Sodium silicate	0.0, 0.6	Cd toxicity	4 days	Rice	Farooq et al., 2016

Seed priming is a technique that has been in use for more than 100 years. Around 2,600 research articles have been published between 2010 and 2022, but only around 50 documents were related to Si priming (e.g., Azeem et al., 2015; Ahmed et al., 2016). Different Si sources used for priming have been summarized in Table 2. In general, for seed priming, the ratio of 1:5 (w/v) seed weight to solution volume was maintained and seeds were dried before being sown (Ali et al., 2021; Chakma et al., 2021). Most of the studies are performed with sodium or potassium silicate. But only at solution pH above 8.5, silicates are the main form of Si in solution, being the monosilicic acid the prevalent form at pH below this (optimal pH for crop cultures). As silicates are highly used, the pH of the priming solution becomes very high, which could probably alter the seed performance, metabolism, and growth after desiccation or the seedlings–plant development. If the pH of the priming solution with silicate is reduced by adding some acid, as hydrochloric acid or others, silicon will precipitate as SiO_2_⋅nH_2_O, so the priming effect is clearly reduced by reducing the concentration of silicon in the priming solution. On the contrary, silicic acid solutions let to adjust different pHs of the priming solution without any precipitation of the priming agent at the optimal range for plant production. Its main problem is the polymerization of the monosilicic acid into polisilicic acid at high concentrations, but concentrations used in plant culture are low enough to avoid it.

## Silicon Priming Effect on Plant Growth Under Non-Stressed Conditions

Although the Si effect is described in the literature to be more relevant under plant stress situations, also beneficial effects of seed priming with this element are described in well-fed plants. Kumaraswamy et al. (2021) tested SiO_2_ (0.01% w/v) and SiO_2_ encapsulated in a chitosan (a cationic amino-polysaccharide)-tripolyphosphate nanomatrix (0.01, 0.04, 0.08, 0.12, 0.16%, w/v) as a slow-release Si source in priming solutions for maize seeds. They have found that seeds primed with 0.04–0.12%, w/v of the nano-Si fertilizer exhibited up to 3.7-fold increased seedling vigor index as compared to SiO_2_ and treatments without Si. The higher index was attributed to enhanced activities of α-amylase and protease to promote remobilization of reserved nutrients (glucose and amino acids) to the growing embryo (Kumaraswamy et al., 2021). Janmohammadi and Sabaghnia (2015) studied the effect of seed soaking in different concentrations of nanosilicon (nSiO_2_) solutions (0.0, 0.2, 0.4, 0.6, 0.8, 1.0, and 1.2 mM) for 8 h on the germination of sunflower. Seed soaking in 0.2 and 0.4 mM solutions significantly reduced the germination days to 50% and improved root length, mean daily germination, seedling vigor index, and final germination percentage. Hameed et al. (2021) primed spring wheat seeds with 20, 40, and 60 mM sodium silicate solutions, for 8 h; after drying, seeds were cultivated for 98 days. In flag leaves of mature wheat plants, they have observed significant increases of the total soluble protein content and the reducing and total sugars with increasing Si concentrations. Moreover, the Si priming treatments significantly increased the CAT and POD activities, and also both hydrolytic (protease and a-amylase) enzymes. This suggested the facilitating role of Si in protein synthesis under optimal conditions. The role of sugars as osmoprotectants and membrane stability providers was also accentuated with Si addition to the media (O’Hara et al., 2013). The enhancement of proteins and sugar content could have assisted in the regulation of metabolic pathways and provided energy and nutrients for the induction of stress tolerance, if appears. Consequently improving yield, plant biomass, and grain weight of wheat plants grown under optimal conditions (Hameed et al., 2021).

Moreover, Sun et al. (2021) tested the effect of two water-soluble Si fertilizers (3-aminopropyl triethoxy silane (pH 5.95) and 3-glycidoxypropyl trimethoxy silane (pH 9.42) synthesized by high-temperature chemical reactions in maize at different concentrations in the soaking solution: 0, 5, 10, 15, 20, and 25 g⋅L^–1^. In this study, Si treatments significantly increased the seed germination, chlorophyll content, osmotic material accumulation, antioxidant activity, and per-plant dry weight of seedlings, and the optimal concentration was 15 g⋅L^–1^. Hatami et al. (2021) have studied the priming of lemon balm (*Melissa officinalis* L.) seeds with SiO_2_ nanoparticles (100 and 500 mg⋅L^–1^). Seeds were soaked in dark at 20°C for 24 h (seed weight: solution ratio, 1.4 g: mL^–1^) and then surface-dried for 2 h and stored at 4°C. They have concluded that Si priming increased plant biomass indices, leaf relative water content, photosynthetic pigments values, total soluble protein, phenolic contents, and essential oil yield. These results suggest that the incorporation of silicon in priming solution enhanced germination and invigoration of the seedling and provides fitted plants to cope with biotic and abiotic stresses and thus contributes to growth and crop yield. The mechanisms described to explain this effect have been related to the alteration of the surface texture of seed coat (testa) (Hatami et al., 2021). These authors using scanning electron microscopy (SEM) images confirmed the rupture of testa by Si priming, although no alteration was detected in control treatments with water. A high porosity degree and large pores, and also a partial disorganization of the surface testa structure, facilitate the entry of water, nutrients, and oxygen into the germinating seed and may explain the Si effect on the future plants. This may also enhance the plant biomass and growth in comparison with untreated plants. Moreover, it has been described that Si increase the relative water content found in plants raised from seeds primed with nSi (Hatami et al., 2021), which was attributed to the Si deposition in leaves, which diminish transpiration rate from leaf surface and significantly contribute to increase photosynthetic pigments content, due to plant erectness.

The most used primers are silicates and SiO_2_, but Si concentrations tested ranged from 0.2 to 60 mM, so further research is required to adjust the most adequate Si source and the concentration to be used. Such parameters could be different depending on plant species, but it is worthwhile to dedicate time and effort to establish optimal conditions for priming with Si according to the benefits already described with this simple and affordable technique.

## Silicon Priming Effect Under Micronutrient Deficiency Stress Conditions

**FIGURE 1:**
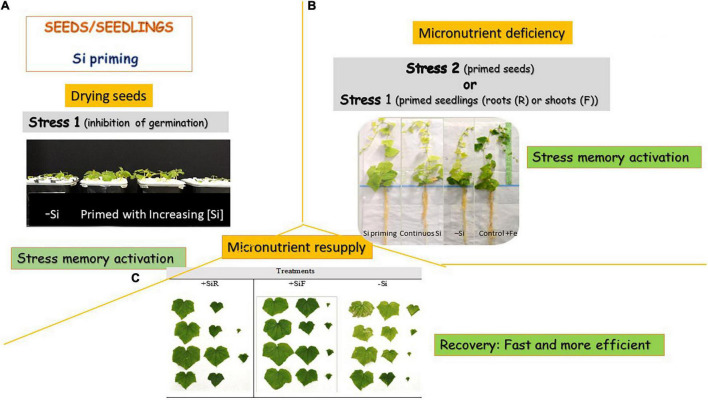
Silicon priming could be done in **(A)** seeds or seedlings or even in plant parts, and in all cases, stress memory is activated by a stress caused by seeds desiccation. When another stress condition appears, such as a micronutrient deficiency **(B)**, plant stress memory is activated again, prevent plants from stress symptoms and accelerate plant recovery **(C)** when stress stops. In this case, the scheme and photos are referred to micronutrient deficiency (Fe) and its resupplying to cucumber plants.

Silicon seed priming has been used to mitigate several stresses (Table 2); however, to our knowledge, it has not been used for micronutrient stress amelioration until now. Although less often, priming is applied also to seedlings or their parts in active growth phases (Sytar et al., 2019). Few papers tested the Si seedling priming effect under micronutrient shortage. In that way, to mitigate Fe chlorosis symptoms, Gonzalo et al. (2013) compared soybean and cucumber seedlings primed with different doses of root Si (Figure 1), with unprimed plants, and with plants with a continuous Si supply. For that purpose, germinated seeds were grown for 2 weeks with a sufficient Fe supply and three Si doses as Na_2_SiO_3_⋅9H_2_O (0.0, 0.5, and 1.0 mM). Then, Fe was removed from the nutrient solution, at the same time, half of the plants of each Si treatment continued with their Si supply for 3 weeks more, and for the other half, Si was eliminated from the nutrient solution (seedling priming). A control with Fe and without Si addition was also studied. For soybean, no differences were observed in SPAD index and leaves dry weight between plants treated with 0.5 mM Si either when this element was applied initially (seedling priming) or continuously during the experiment. Both presented intermediate SPAD values in between plants growing with Fe and without Si (positive control plants) and plants growing without both Fe and Si (negative control plants), and leaves dry weight were similar to plants with an optimal Fe supply. However, concerning the stem’s dry weight and length, only the initial addition of 0.5 mM of Si showed similar data to plants treated with an optimal Fe supply. Plants primed with 1.0 mM Si showed an enhancement of Fe accumulation in the roots compared to the others, although the total Fe concentration in plants was similar for all the deficient treatments. This fact may explain the lowest efficacy of this treatment compared to the primed 0.5 mM Si one. These authors concluded that for soybean, a priming treatment of 2 weeks with 0.5 mM Si will contribute to better coping with Fe deficiency symptoms than a continuous Si supply or no Si addition. This could be attributed to that Si priming-induced physiological responses that allow the plant to give a more efficient and rapid answer to the imposed Fe deficiency stress. Becker et al. (2020) reported that the cause of this quick answer was the Fe uptake reduction caused by the Si-mediated apoplastic obstruction in the roots and the subsequent onset of Fe deficiency responses (the root Fe-homeostasis-related genes were upregulated), even when Fe was given to plants at an optimal level. Therefore, when plants were submitted to iron deficiency, primed plants, which have already activated the strategies to mitigate Fe chlorosis, are ready to fight against Fe shortage in the media. Data obtained by Carrasco-Gil et al. (2018) in rice support these findings. On the other hand, results obtained after priming cucumber plants with 0.5 and 1.0 mM Si to cope with Fe deficiency showed similar severe chlorosis symptoms to unprimed plants and plants grown with a continuous Si supply (Gonzalo et al., 2013). However, these authors observed that plants primed with 0.5 mM Si showed a relevant enhancement in growth parameters. So clearly, the effect of Si priming was related to plant species tested. The link between the Si transport system and its accumulation could give a plant classification into active, passive, and rejective. In the active uptake system of Si, Si absorption is mediated by both influx and efflux transporters of Si; Lsi1 and Lsi2, and both of them show polar localization (e.g., rice) (Mitani-Ueno and Ma, 2021). In the passive transport system (employed by plants having intermediate Si accumulation such as cucumber, in which CsLsi1 and CsLsi2 have been partially characterized), most of the Si transporters do not show polar localization at the cortex cells except CsLsi1, and Lsi1 and Lsi2 in these plant species are not localized at the same cell, which results in low efficiency in Si uptake (Mitani-Ueno and Ma, 2021). The rejective uptake system is used by non-Si accumulators such as soybean, in which other transporters homolog of Lsi1 and Lsi2 have been described (GmNIP2-1 and GmNIP2-2) (Deshmukh et al., 2013), which seems to be less effective than the previously described. Beneficial effects of Si are usually obvious in plants that accumulate high levels of Si in their shoots, such as rice or sugarcane, so the beneficial effect of Si in cucumber should be more visible than in soybean. However, it is considered that Si promotes apoplastic obstruction, which limits Fe and other micronutrient absorption in the plant, so the more Si was absorbed the less Fe uptake. When this element is deficient in the media, soybean plants that are supposed to absorb less amount of Si showed the highest benefits from its addition; but in cucumber, the higher Si absorption, although benefit several growth parameters, may induce the Fe apoplastic obstruction, and activating the Fe deficiency strategy with the corresponding energy loss, which makes its benefit less clear. Gonzalo et al. (2013) indicated that in cucumber, the primed plants, either with 0.5 or 1.0 mM Si, significantly decreased the pH of the nutrient solution, from 7.5 until pH 4.5, after 14 days of -Fe culture, but after 21 days of Fe deficiency, only seedlings primed with 1.0 mM Si gave a pH value in the nutrient solution of 5.9 (the initial pH of the nutrient solution was 7.5). This showed the onset of the strategies to cope with Fe deficiency, as the release of acidic compounds to solubilize Fe in the rhizosphere, and the finite duration of them. Priming with Si seemed to maintain Fe deficiency strategy more time than a continuous Si supply or the absence of this element, but future research is required to confirm this fact. The soybean did not decrease the pH of the nutrient solution. Likewise, Pascual et al. (2016) tested the effect of initial or continuous Si supply in soybean Zn-deficient seedlings. Three Si doses were tested: 0.0, 0.5, and 1.0 mM under Zn limiting conditions. The initial addition of 0.5 mM of Si to the nutrient solution led to an enhancement of plant growth, Zn and Si content in leaves, and higher storage of Zn in the root apoplast. The results suggest that primed seedlings with 0.5 mM Si enhanced the mitigation of Zn deficiency symptoms. To the author’s knowledge, no further Si seedling prime experiments have been done, but the ones presented here suggest a very promising tool in nurseries to get plants more prepared to cope with Fe or Zn deficiency situations.

## Recovery Experiments With Silicon Addition

Very few papers are related to plant recovery memory (plant memory after a stress situation) dealing with Si application. For example, Si-mediated recovery from hyperhydricity was studied in 4-week-old hyperhydric shoots of carnation (*D. caryophyllus* L.) plants in a growth media supplemented with 0.0, 1.8 mM, or 3.6 mM of potassium silicate (Soundararajan et al., 2017). Hyperhydricity (excess of water) causes severe problems during *in vitro* propagation, organogenesis, and acclimatization of carnation, which is one of the major floricultural crops, mainly used as a cut flower and potted plant worldwide. After 2 weeks of culture, 20, 44, and 36% of hyperhydric shoots were recovered in 0.0, 1.8, and 3.6 mM Si treatments, respectively. Shoots in control possessed higher lipid peroxidation rate and damaged stomata were detected in the control without Si. Furthermore, Si upregulated 17 protein spots at 1.8 mM Si treatment and 10 protein spots at 3.6 mM of Si when compared to Si untreated plants. The proteins that have been identified were involved in several processes such as oxide-reduction reactions, ribosomal binding, hormone–cell signaling, photosynthesis, and defense. These results showed that Si was directly involved in the acceleration of shoots recovery from hyperhydricity (Soundararajan et al., 2017). The influence of Si in metal toxicity recovery was also studied. At the age of 38 days, rice plants were stressed with 10 μM Cd added to the nutrient solution for 8 days, and then, the silicon treatments (0.0 or 0.6 mM Si) were introduced 4 days after Cd stress using a sodium silicate (Na_2_SiO_3_) solution which was maintained for 4 days more (Farooq et al., 2016). In this experiment, Si remarkably contributes to recovering plants from the Cd toxicity, as reflected in plant growth increase and the photosynthetic activity recovery within 48 h following Si supply and the partial reversion of the deregulation of nutrient homeostasis caused by Cd. The transcriptional response to Cd was mostly reversed following Si supply as several proteins–enzymes as phytochelatin synthase 1 and the transcription factor genes whose transcript levels were highly activated in the Cd stressed roots were downregulated in the presence of Si (Farooq et al., 2016).

Finally, the Si effect on plant recovery from micronutrient deficiencies has also been investigated (Figure 1). Silicon addition as silicic acid at a 1.5 mM Si concentration (applied to roots or shoot) was evaluated on cucumber plants recovery exposed to fluctuations in stress–recovery Fe regime (Fe sufficiency followed by Fe deficiency and, in turn, by Fe resupply) (Hernandez-Apaolaza et al., 2020). Si-treated plants, either when this element was added to the root or the leaves, showed a more effective and quick plant recovery after the Fe deficiency period compared to the untreated plants. However, the SPAD index increment after resupply was higher and the ROS concentration lower when Si was supplied to the roots than to the shoot, which indicates that these plants had recovered from the chlorosis faster than the others. It was suggested that the extra-activation of the strategies to cope with Fe deficiency promoted by Si in the roots, due to the apoplastic obstruction theory (Coskun et al., 2019), may cause this better recovery. However, there is another hypothesis that may explain this behavior. As mentioned above, several stress memories resulted in a rapid increase in endogenous ABA content. ABA plays a key role in plant stress resistance and changed the postresponse gene type into the memory gene type, which probably enhances plant recovery (Qin et al., 2021). The higher ABA concentration in the shoots of the cucumber plants treated with root Si was in accordance with this theory. Meanwhile, the foliar addition of Si did not show any differences in this hormone. Plant recovery was also correlated with an increase in the endoreplication cycle when Si was applied to the roots; this mechanism prevents plants from damage and then facilitates their recovery from stress. It is known that ABA inhibits cell division, so cells are devoted to the endoreplication cycle. The higher ABA concentration that promotes the switch on of the endoreplication cycle may explain the quick recovery from Fe deficiency of root Si treated plants. Likewise, Lozano-González et al. (2021) studied the effect of Si supply (1.5 mM as silicic acid applied to roots or shoots) on cucumber plants’ recovery from Zn deficiency. They concluded that the Si application reduced plant recovery time. In that case, foliar application of Si showed faster improvement in SPAD index, higher weight recovery, and a significant decrease in ROS quantity, but this effect was slightly lower when Si was applied through the root.

The state of the art today indicates that using Si to accelerate and improve plant recovery from different stresses such as hyper hydricity, metal toxicities or deficiencies are very promising tools. Silicon addition may not cause toxicity itself, but the Si source used and its concentration needs to be addressed for each specific recovery, and also the application form (roots of foliar sprays) and the consequences on fruit quality and shelf life.

## Perspectives

The increasing amount of published papers dealing with plant memory may open a new research field to cope with plant stresses in a smart form that takes profit from very simple management practices, such as seed or seedling priming to ameliorate yield losses in various crops. It is especially interesting considering the global climate change in which plants have to cope with higher temperatures, drought, salinity, and other stresses as nutrient imbalances. Plant stress memory not only contributes to dealing with the stress itself but also makes plant recovery after it in a more fast and efficient way. Although several mechanisms have been studied to explain the effect of primming in stress memory and plant recovery, being the histone marks in chromatin the most studied, there is an increasing necessity of knowing how primers interact with the plants. Likewise, it is necessary to define the amount of them and the time needed to obtain the desired beneficial effect or a crosseffect for various biotic or abiotic stresses at the same time. Several priming agents are tested for different stresses, most of them with great success, but several questions are still open. For example, the beneficial effect of their application could be observed only in the stress plants or through different generations? It happens in all crops and for all types of stresses? In which crops it finishes when stress finishes? What happens with recurrent stresses (a normal situation in drought and high-temperature episodes)? May priming agents cause negative reactions? Is it better to use seed or seedling priming for specific stress? All these features and more need to be addressed to maximize the advantages of plant memory, which like vaccination in humans and animals may create a plant physiological state to prepare to fight against stresses but minimize the energy expenses.

There are four accepted and common ways of silicon addition to the plants which are silicon addition to the soil, silicon added through the nutrient solution in hydroponics, add as foliar–fruit sprays, and the less-studied Si seed–seedling priming. Silicon priming is an economical non-expensive and easy to handle way to promote plant growth, fight against different biotic and abiotic stresses in plants, and promote plant recovery after stress. In recent years, prospective research works have been done about Si application as a primer on alleviation of the effects of several environmental biotic and abiotic stresses. But it is expected that novel research works will be done regarding this issue.

## Author Contributions

LH-A conceived, designed the review, wrote the manuscript, and corrected the manuscript, read and approved the manuscript.

## Conflict of Interest

The author declares that the research was conducted in the absence of any commercial or financial relationships that could be construed as a potential conflict of interest.

## Publisher’s Note

All claims expressed in this article are solely those of the authors and do not necessarily represent those of their affiliated organizations, or those of the publisher, the editors and the reviewers. Any product that may be evaluated in this article, or claim that may be made by its manufacturer, is not guaranteed or endorsed by the publisher.
